# Short and long-term readmission after major emergency abdominal surgery: a prospective Danish study

**DOI:** 10.1007/s00068-023-02352-3

**Published:** 2023-08-30

**Authors:** Lív í Soylu, Dunja Kokotovic, Ismail Gögenur, Sarah Ekeloef, Jakob Burcharth

**Affiliations:** 1 Department of Gastrointestinal and Hepatic Diseases, Emergency Surgical Research Group (EMERGE), Copenhagen University Hospitals - Herlev and Gentofte, Borgmester Ib Juuls Vej 1, 2730 Herlev, Denmark; 2 Center for Surgical Science, Zealand University Hospital, Køge, Denmark

**Keywords:** Emergency surgery, Abdominal surgery, Readmission, Long-term outcomes, Rehabilitation

## Abstract

**Purpose:**

Major emergency abdominal surgery is associated with severe in-hospital complications and loss of performance. After discharge, a substantial fraction of patients are readmitted emergently; however, limited knowledge exists of the long-term consequences. The aim of this study was to examine the risks and causes of short-term (30-day) and long-term (180-day) readmission among patients undergoing major emergency abdominal surgery.

**Methods:**

This study included 504 patients who underwent major emergency abdominal surgery at the Zealand University Hospital between March 1, 2017, and February 28, 2019. The population was followed from 0 to 180 days after discharge, and detailed readmission information was registered. A Cox proportional hazards model was used to examine the independent risk factors for readmission within 30 and 180 days.

**Results:**

From 0 to 30 days after discharge, 161 (31.9%) patients were readmitted emergently, accumulating to 241 (47.8%) patients within 180 days after discharge. The main reasons for short-term readmission were related to the gastrointestinal tract and surgical wounds, whereas long-term readmissions were due to infections, cardiovascular complications, and abdominal pain. Stomal placement was an independent risk factor for short-term readmission, whereas an ASA score of 3 was a risk factor for both short-term and long-term readmission.

**Conclusion:**

Close to 50% of all patients who underwent major emergency abdominal surgery had one or more emergency readmission within 180 days of discharge, and these data points towards the risk factors involved.

**Supplementary Information:**

The online version contains supplementary material available at 10.1007/s00068-023-02352-3.

## Introduction

Major emergency abdominal surgery is associated with a high postoperative morbidity and mortality risk. Five to 15% of all patients undergoing abdominal emergency surgery are readmitted within the first 30 days after discharge [1–5]. For patients undergoing *major* emergency abdominal surgery, the reported number of 30-day readmission varies between 9 and 18% [6–8], however, this has not been thoroughly examined.

Both surgical urgency and invasiveness are potential risk factors adversely affecting postoperative recovery [9–11]; leaving patients undergoing major emergency abdominal surgery especially vulnerable to poor outcomes. Current literature mainly focuses on minor emergency abdominal surgery, and research beyond the immediate postoperative period is sparse.

The main reasons for short-term readmission after abdominal surgery are wound infections and gastrointestinal complaints [1, 2, 12], and postoperative complications before discharge seem to increase the risk of readmission [13]. One study found that readmissions were mainly due to complications that occurred after discharge rather than an exacerbation of in-hospital complications [14]. This finding supports that the duration at which patients risk complications after major surgery exceeds the immediate postoperative period. However, readmissions beyond 30 days after discharge have rarely been examined, and little is known about the long-term consequences of major emergency abdominal surgery.

This study aimed to examine the risks and causes of short-term (30-days) and long-term (180-days) readmissions among patients undergoing high-risk major emergency abdominal surgery in a Danish setting.

## Methods

### Study population and index admission

The Optimizing Major Emergency Abdominal Surgery (OMEGA) cohort was established at the Department of Surgery, Zealand University Hospital, on 1 March 2017, and its inclusion is still ongoing. All patients (≥ 18 years of age) undergoing major emergency abdominal surgery, open or laparoscopic (including emergency reoperations after elective surgery) due to intra-abdominal pathologies such as bowel perforation, ischemia, abscess, bleeding, obstruction, or fascial dehiscence, were included. Patients undergoing emergency surgery due to trauma and emergency hernia repair without bowel resection, as well as minor emergency surgery due to diseases of the appendix, gallbladder, liver, spleen, kidney, or pancreas, were excluded [15]. All patients registered in the OMEGA cohort from March 1, 2017, to February 28, 2019, were included in this study. In case of multiple registrations per patient during the inclusion period (due to renewed high-risk surgery), the earliest registration was used as the index admission.

The index admission was defined as the period from the first day of admission until the final date of discharge, regardless of entering or discharging department; thus, patients transferred to other departments in continuation of the surgical admission were considered finally discharged when they were discharged from the non-surgical admission and left the hospital.

### Assessment of emergency readmissions

All emergency hospital contacts from 0 to 180 days after discharge were identified from the patient records. The contact was registered as readmission when it included a doctor’s physical examination and evaluation, regardless of the length of stay or hospital department. The records were reviewed, and each readmission was registered independently; thus, patients with more than one readmission during the follow-up period of 180 days were registered equally numerous times, even if the repeated emergency readmission was due to the same diagnosis. The primary diagnosis of each emergency readmission was registered according to the International Classification of Diseases (ICD)-10 and categorized according to organ system into readmissions related to (1) skin, wounds, and incisions; (2) the gastrointestinal tract; (3) thromboembolic events, or the cardiovascular or pulmonary system; (4) electrolyte and fluid balance; (5) infectious diseases; (6) cerebral diseases, and (7) other reasons (social reasons, psychiatric diseases, urinary tract). Furthermore, each emergency readmission/contact was evaluated for relevance to the index admission, and when in doubt, the relevance was assessed and discussed within the author group. The readmission/contact was excluded when the relevance was interpreted as definitely unrelated to the index admission.

### Variables

Detailed information on baseline characteristics and pre-, intra-, and postoperative factors were retrieved from the OMEGA cohort registry. Postoperatively, all patients received a performance evaluation from a physiotherapist with special training in evaluating postoperative patients. Patients who had experienced loss of performance about their current disease/admission were discharged to their own home or a rehabilitation facility with planned physiotherapy. The variable “destination at discharge” describes the discharge destination and information on planned physiotherapy and is divided into four categories (1) home without planned physiotherapy, (2) home with planned physiotherapy, (3) discharge to rehabilitation facility, and (4) other discharge (nursing home, hospice).

### Statistical analysis

The distribution of continuous data was assessed by visual inspection of histograms. Categorical data are presented as the number of cases and percentages. For continuous variables (age, body mass index (BMI), and length of stay (LOS) of index admission), the median and interquartile (IQR) ranges were calculated, and the Mann–Whitney *U* test was used for comparison. Differences in categorical variables (binary and polytomous variables) between patients with and without emergency readmissions were analyzed using Pearson’s chi-square test and Fisher’s exact *t*-test. Furthermore, all chi-squared tests were adjusted for multiple comparisons using the Benjamini–Hochberg method.

All emergency readmissions (0–180 days after discharge) were registered and divided into three categories: (1) short-term readmission (0–30 days after discharge), (2) intermediate-term readmission (31–90 days after discharge), and 3) long-term readmission (91–180 days after discharge).

According to readmission status, Kaplan Meier survival analysis was performed for 180-day mortality.

A Cox proportional hazards regression was performed to identify independent risk factors for 30- and 180-day readmission to estimate hazard ratios (HRs) and 95% confidence intervals (CIs). Days from discharge were used as the underlying time scale, and patients were followed until 180 days after discharge. Patients likely to be lost to follow-up (tourists) were excluded. Furthermore, patients who had a missing discharge date due to registration error or extreme prolonged admission were excluded.

Based on current literature, relevant clinical variables where included in the analyses: sex, age (< 60, 60–69, 70–79 or ≥ 80 years of age), performance status (0–1 or ≥ 2), ASA-score (1–2, 3 or 4–5), active smoking (yes/no), BMI (< 18.5, 18.5–24.9, 25–29.9, or ≥ 30), active cancer (yes/no), index procedure (upper gastrointestinal surgery, surgery without resection, surgery with bowel resection and anastomosis, surgery with bowel resection and stoma or another type of surgery), any postoperative complication during the index admission (yes/no), reoperation before discharge (yes/no), discharge destination from index admission (home, home with physiotherapy, rehabilitation facility or another facility), discharge from non-surgical departments (yes/no), and LOS of index admission (continuous variable). ASA score was used as a proxy for comorbidities. Furthermore, we included active cancer as an independent variable because of the high risk of readmission in this group of patients, both due to active cancer and cancer treatments.

All analyses were performed using SAS software, version 9.4. SAS Institute Inc., Cary, NC, USA.

The study was approved by the Danish Data Protection Agency (no: REG-042–2017). The study did not qualify for ethics approval by Danish law as no intervention was carried out.

## Results

**Fig. 1:**
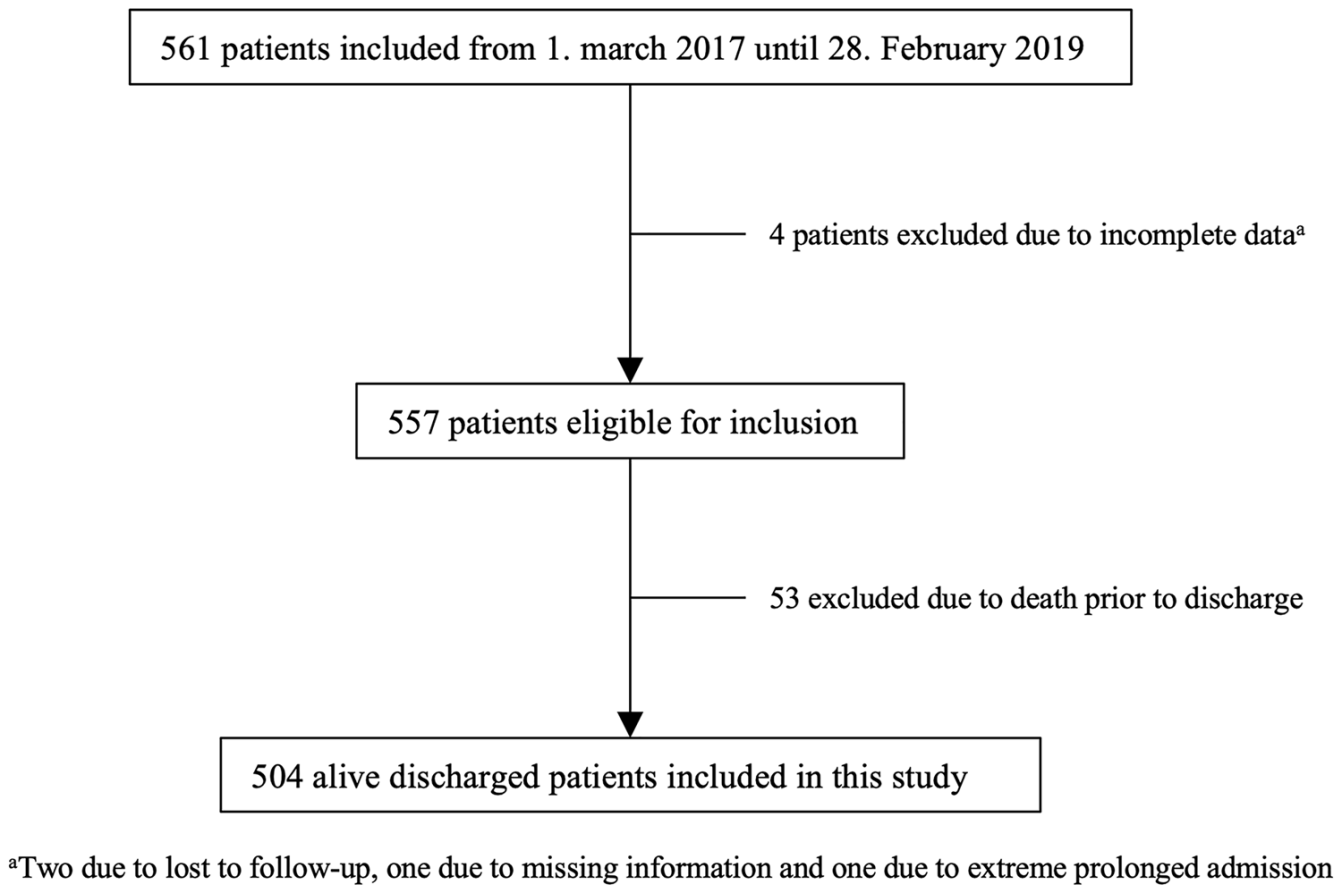
Identification of the study population from the Optimizing Major Emergency Abdominal surgery (OMEGA) cohort

We evaluated 561 and analysed 504 patients (Fig. 1). The 504 included patients had a total of 460 emergency readmission, of which 27 were excluded as they were not considered relevant to the index admission (due to renewed planned abdominal surgery between discharge and readmission, no doctor’s examination before discharge, or obvious external factors such as unrelated accidents, self-inflicted removal of catheters/tubes, and side effects of oncologic chemotherapy), leaving a total of 433 readmissions. The first 201 (46.4%) emergency readmissions occurred within 30 days after discharge, adding up to 329 (76.0%) readmissions 90 days after discharge, and the last 104 (24.0%) readmissions occurred from 91 to 180 days after discharge.

A total of 241 (47.8%) patients had one or more emergency readmission during the follow-up period from 0 to 180 days after discharge, of whom 161 (66.8%) had their first readmission during the first 30 days. Additionally, 47 (19.5%) patients had a readmission between days 31–90, adding up to 208 (86.3%) patients in the first 90 days. Meanwhile, 33 (13.7%) patients had a readmission between days 91–180 after discharge.

### Baseline characteristics and postoperative course

The median LOS among patients who had no readmissions within 180 days after discharge was seven days, as opposed to nine days in patients who had readmissions. Furthermore, LOS was significantly associated with an increased likelihood of readmission (*p* = 0.02) (Table 1), as well as pulmonary comorbidity (*p* = 0.02) and ASA score (*p* = 0.012) (Table 2).

**Table 1:** Pre- and intraoperative characteristics according to emergency readmission status

	Readmission (no)	Readmission (yes)	*p*-value ^a^
Total, no	263	241	–
Male, no (%)	134 (51.0)	124 (51.5)	0.98
Age, no (%)			0.55
< *60*	84 (31.9)	69 (28.6)	
*60–69*	62 (23.6)	53 (22.0)	
*70–79*	77 (29.3)	79 (32.8)	
≥ *80*	40 (15.2)	40 (16.6)	
*Median (IQR)*	67 (55–76)	69 (57–77)	
Married / cohabiting, no (%)	159 (60.5)	146 (60.6)	0.99
Current smoker, no (%)	55 (21.9)	59 (24.5)	0.55
Alcohol in excess, no (%) ^b^	31 (11.8)	20 (8.3)	0.39
BMI, median (IQR)	25.0 (22.2–28.7)	24.8 (21.8–29.6)	0.98
**Comorbidities, no (%)**			
Abdominal comorbidity ^c^	39 (14.8)	52 (21.6)	0.18
Cardiac comorbidity	130 (49.4)	126 (52.3)	0.66
*Arterial hypertension*	99 (37.6)	77 (32.0)	0.39
*Hypercholesterolemia*	12 (4.6)	18 (7.5)	0.39
*Arrhytmias*	27 (10.3)	27 (11.2)	0.82
*Ischemic heart disease*	22 (9.1)	20 (7.6)	0.66
*Congestive heart failure*	9 (3.4)	11 (4.5)	0.66
*History with cerebral ischemia*	10 (3.8)	21 (8.7)	0.10
*Other cardiac comorbidity *^*c*^	20 (7.6)	26 (10.8)	0.41
Pulmonal comorbidity	21 (8.0)	43 (17.8)	0.02
*COPD *^*d*^	19 (7.2)	31 (12.9)	0.14
*Other pulmonal comorbidity *^*c*^	4 (1.5)	16 (6.6)	0.05
Chronic kidney disease	11 (4.2)	18 (7.5)	0.34
Diabetis Mellitus	25 (9.5)	29 (12.0)	0.55
Active cancer	31 (11.8)	41 (17.0)	0.29
Neurological comorbidity	14 (5.3)	16 (6.6)	0.66
Psychiatric comorbidity	15 (5.7)	22 (9.1)	0.39
WHO Performance Status			0.09
*0*	152 (57.8)	114 (47.3)	
*1*	78 (29.7)	76 (31.5)	
≥ *2*	33 (12.6)	51 (21.2)	
ASA Physical Status Classification System			0.012
*1–2*	181 (68.8)	123 (51.0)	
*3*	75 (28.5)	108 (44.8)	
*4–5*	7 (2.7)	10 (4.2)	
**Surgical procedure **^**e**^**, no (%)**			0.14
Upper gastrointestinal surgery	27 (10.3)	22 (9.1)	
Surgery without bowel resection	135 (51.3)	110 (45.6)	
Bowel resection w/ anastomosis	29 (11.0)	20 (8.3)	
Bowel resection w/ stoma	59 (22.4)	83 (34.4)	
Other ^f^	13 (4.9)	6 (2.5)	
**Type of procedure, no (%)**			0.49
Laparoscopic	38 (14.5)	24 (10.0)	
Laparoscopic converted to open	47 (17.9)	41 (17.0)	
Open	178 (67.7)	176 (73.0)	

^a^*p*-value adjusted with Benjamini Hochberg procedure

^b^More than 21 or 11 units of alcohol per week for men and women, respectively

^c^Characterised as other due to the minor incidence of the event

^d^Chronic obstructive pulmonary disease (COPD)

^e^Open and laparascopic procedures

^f^Splenectomies, appendectomies, cholecystectomies and patients who were transferred for final surgery

**Table 2:** Postoperative characteristics according to emergency readmission status

	Readmission (no)	Readmission (yes)	p-value ^a^
Reoperation prior to discharge, no (%)	52 (19.8)	64 (26.6)	0.24
Postoperative ICU admission, no (%)	32 (12.2)	33 (13.7)	0.72
Transferred to another department, no (%)	26 (9.9)	39 (16.2)	0.14
Length of stay, median (IQR)	7 (14–4)	9 (17–5)	0.02
Destination at discharge			0.10
* Home without rehabilitation*	189 (71.9)	147 (61.0)	
* Home with rehabilitation*^*b*^	40 (15.2)	63 (26.1)	
* Rehabilitation facility*	23 (8.8)	23 (9.5)	
* Other *^*c*^	11 (4.2)	8 (3.3)	
**Postoperative complications**			
Any postoperative complication, no (%)	157 (59.7)	170 (70.5)	0.08
Clavien-Dindo classification ^d^			0.06
* 1*	9 (3.4)	7 (2.9)	
* 2*	40 (15.2)	55 (22.8)	
*≥ 3*	108 (41.1)	108 (44.8)	
**Postoperative complications according to diagnosis**			
Overall no. of complications, median (IQR)	2 (4–1)	3 (5–1)	0.55
Wound complications	15 (5.7)	22 (9.1)	0.36
Overall intraabdominal complications	109 (41.4)	127 (52.7)	0.08
* Facial dehiscence*	14 (5.3)	17 (7.1)	0.58
* Mechanical obstruction*	10 (3.8)	11 (4.6)	0.77
* Prolonged paralysis*	30 (11.4)	37 (15.4)	0.39
* Intraabdominal abscess*	12 (4.6)	15 (6.2)	0.58
* Bleeding and/or transfusion*	17 (6.5)	23 (9.5)	0.40
* Other abdominal complications *^*e*^	12 (4.6)	16 (6.6)	0.53
Overall cardiologic complications	48 (18.3)	44 (18.3)	0.99
* Arrythmias*	17 (6.5)	16 (6.6)	0.99
* MINS*^*f*^	28 (10.7)	26 (10.8)	0.99
* Other cardiac complications *^*e*^	7 (2.7)	7 (2.9)	0.96
Overall pulmonal complications	73 (27.8)	82 (34.0)	0.36
* Respiratory failure*	20 (7.6)	11 (4.6)	0.39
* Pneumonia*	48 (18.3)	57 (23.7)	0.36
* Other pulmonal complication *^*e*^	12 (4.6)	23 (9.5)	0.13
Overall infectious complications	30 (11.4)	39 (16.2)	0.35
* Sepsis*	16 (6.1)	20 (8.3)	0.54
* Urinary tract infection*	5 (1.9)	9 (3.7)	0.41
* Other infections*	15 (5.7)	18 (7.5)	0.58
Thromboembolic complications	5 (1.9)	2 (0.8)	0.53
Renal complications	26 (9.9)	33 (13.7)	0.39
Neurological complications	14 (5.3)	16 (6.6)	0.66
Electrolyte and fluid imbalances	46 (17.5)	46 (19.1)	0.75
Parenteral nutrition	58 (22.1)	79 (32.8)	0.06

^a^p-value adjusted with Benjamini Hochberg procedure

^b^Home-based physiotherapy

^c^Nursing home or hospice

^d^The complication with the highest Clavien-Dindo score was used

^e^Characterised as other due to the minor incidence of the event

^f^Myocardial injury after non-cardiac surgery

Patients discharged with planned physiotherapy more often had emergency readmissions than those discharged without planned physiotherapy; however, the finding was not statistically significant.

### Causes of emergency readmissions

The median time to the first emergency readmission was 13 days (IQR, 5–13 days), and the median number of readmissions was 1 (IQR = 1–2). The majority of patients’ first readmissions were at the general surgical department (48.5%), followed by the internal medicine department (20.3%), emergency department (14.9%), cardiological department (4.1%), and other departments (9.5%). The majority reasons for emergency readmission are presented in Table 3.

**Table 3:** Emergency readmissions according to diagnosis

	Total^a^	(%)	0–30 days^b^	(%)	31–90 days^b^	(%)	91–180 days^b^	(%)
**Total no. of emergency readmissions**	**433**	–	**201**	–	**128**	–	**104**	–
Skin, wounds and incisions	59	(14)	47	(23)	6	(5)	6	(6)
* Hematoma, seroma, wound rupture*	*37*	*(9)*	*33*	*(16)*	*2*	*(2)*	*2*	*(2)*
* Abscess, fistula or skin infection*	*22*	*(5)*	*14*	*(7)*	*4*	*(3)*	*4*	*(4)*
Gastrointestinal	182	(42)	84	(42)	52	(41)	46	(44)
* Fascial dehiscence*	*6*	*(1)*	*5*	*(2)*	*1*	*(1)*	*0*	*(0)*
* Anastomotic leak*	*2*	*(0)*	*2*	*(1)*	*0*	*(0)*	*0*	*(0)*
* Mechanical obstruction*	*18*	*(4)*	*6*	*(3)*	*4*	*(3)*	*8*	*(8)*
* Prolonged paralysis*	*21*	*(5)*	*11*	*(5)*	*6*	*(5)*	*4*	*(4)*
* Postoperative intraabdominal abscess*	*7*	*(2)*	*5*	*(2)*	*2*	*(2)*	*0*	*(0)*
* Constipation*	*23*	*(5)*	*8*	*(4)*	*7*	*(5)*	*8*	*(8)*
* Hepatic failure*	*7*	*(2)*	*1*	*(0)*	*1*	*(1)*	*5*	*(5)*
* Gastric or duodenal ulcers*	*5*	*(1)*	*2*	*(1)*	*2*	*(2)*	*1*	*(1)*
* Vomiting*	*8*	*(2)*	*4*	*(2)*	*3*	*(2)*	*1*	*(1)*
* Abdominal pain*^*c*^	*44*	*(10)*	*21*	*(10)*	*10*	*(8)*	*13*	*(13)*
* Stomal problems*	*10*	*(2)*	*4*	*(2)*	*6*	*(5)*	*0*	*(0)*
* Short bowel syndrome*	*14*	*(3)*	*5*	*(2)*	*5*	*(4)*	*4*	*(4)*
* High-output stoma*	*17*	*(4)*	*10*	*(5)*	*5*	*(4)*	*2*	*(2)*
Cardiovascular, thromboembolic and pulmonal	32	(7)	9	(4)	12	(9)	11	(11)
* Cardiac arrhythmias*	*6*	*(1)*	*3*	*(1)*	*1*	*(1)*	*2*	*(2)*
* Acute myocardial infarction*	*2*	*(0)*	*1*	*(0)*	*1*	*(1)*	*0*	*(0)*
* Congestive heart failure*	*3*	*(1)*	*0*	*(0)*	*2*	*(2)*	*1*	*(1)*
* Thrombosis or thrombophlebitis*	*8*	*(2)*	*1*	*(0)*	*3*	*(2)*	*4*	*(4)*
* Pulmonary embolism*	*5*	*(1)*	*2*	*(1)*	*1*	*(1)*	*2*	*(2)*
* Chest pain*^*c*^	*4*	*(1)*	*0*	*(0)*	*2*	*(2)*	*2*	*(2)*
* Other*^*d*^	*4*	*(1)*	*2*	*(1)*	*2*	*(2)*	*0*	*(0)*
Electrolyte and fluid balance	20	(5)	11	(5)	3	(2)	6	(6)
* Volume depletion*	*15*	*(3)*	*8*	*(4)*	*1*	*(1)*	*6*	*(6)*
* Other*^*d*^	*5*	*(1)*	*3*	*(1)*	*2*	*(2)*	*0*	*(0)*
Infectious	80	(18)	25	(12)	35	(27)	20	(19)
* Pneumonia*	*22*	*(5)*	*7*	*(3)*	*5*	*(4)*	*10*	*(10)*
* Urinary tract infection*	*21*	*(5)*	*8*	*(4)*	*9*	*(7)*	*4*	*(4)*
* Sepsis*	*6*	*(1)*	*2*	*(1)*	*2*	*(2)*	*2*	*(2)*
* Other infections*^*e*^	*31*	*(7)*	*8*	*(4)*	*19*	*(15)*	*4*	*(4)*
Cerebral	19	(4)	5	(2)	9	(7)	5	(5)
* Seizures*	*5*	*(1)*	*2*	*(1)*	*3*	*(2)*	*0*	*(0)*
* Cerebral ischemia*	*4*	*(1)*	*0*	*(0)*	*2*	*(2)*	*2*	*(2)*
* Dizziness*	*6*	*(1)*	*1*	*(0)*	*3*	*(2)*	*2*	*(2)*
* Disorientation*	*4*	*(1)*	*2*	*(1)*	*1*	*(1)*	*1*	*(1)*
Other	41	(9)	20	(10)	11	(9)	10	(10)
* Anaemia*	*6*	*(1)*	*2*	*(1)*	*3*	*(2)*	*1*	*(1)*
* Crisis reaction and depression*	*4*	*(1)*	*2*	*(1)*	*1*	*(1)*	*1*	*(1)*
* Problematic home situation*	*5*	*(1)*	*3*	*(1)*	*0*	*(0)*	*2*	*(2)*
* Trip-related falls in the elderly*^*f*^	*13*	*(3)*	*5*	*(2)*	*5*	*(4)*	*3*	*(3)*
* Other*^*d*^	*13*	*(3)*	*8*	*(4)*	*2*	*(2)*	*3*	*(3)*

^a^All postdischarge emergency readmissions from 0 to 180 days

^b^Postdischarge emergency readmissions stratified according to no. of days from discharge to readmission date

^c^Pain with no evident organic reason

^d^Characterised as other due to the minor incidence of the event

^e^Erysepilas, neutropenic fever, acute sinusitis, cholecystitis, cholangitis, pancreatitis, spontaneous bacterial peritonitis, diverticulitis, enterocolitis due to clostridium dificile, pleural empyema and infection due to central venous catheters

^f^65 years and older

During the total follow-up period (0–180 days after discharge), 37 (15.4%) patients had one or more emergency abdominal procedures performed (including open abdominal surgery, laparoscopic abdominal surgery, or superficial debridement of wound).

### Independent risk factors for any emergency readmission

In a Cox proportional hazard regression model ASA-score = 3 was an independent risk factor for emergency readmission within both 30 and 180 days after discharge (hazard ratio (HR) = 1.61, 95% confidence interval (CI) = 1.11–2.33, *p* = 0.013 and HR = 1.44, 95% CI = 1.06–1.95, *p* = 0.002, respectively). Furthermore, stomal placement during the index procedure was an independent risk factor of 30-day readmission HR = 1.27. 95% CI = 1.02–2.23, *p* = 0.04. Independent risk factors are presented in Table S1 in the supplemental material.

### Mortality

**Fig. 2:**
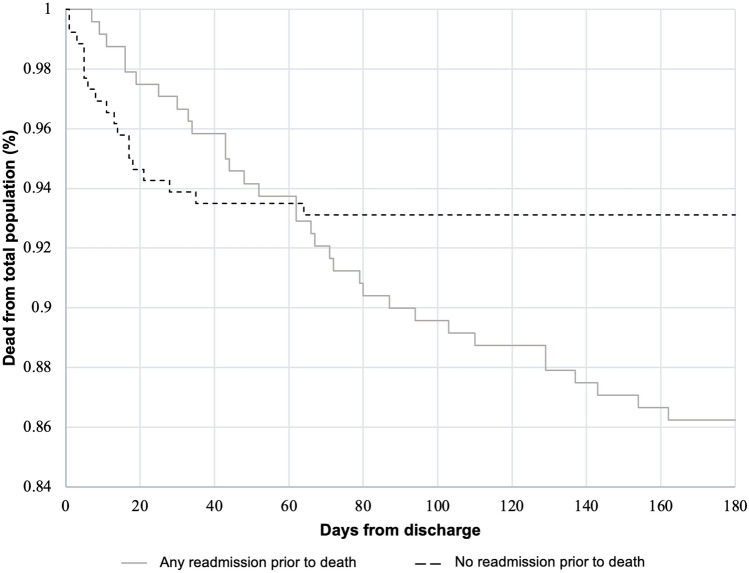
180-day mortality according to emergency readmission status

During follow-up, 51 (10.1%) patients died within 180 days of discharge. Among the patients who died, the median time to readmission was 7.5 days (IQR = 4–18), and the median number of readmissions was 2 (IQR 1–3). The 30-day mortality was 4.8%, and patients who died during the first 30 days were less likely to have been readmitted (3.3 vs. 6.1%, *p* = 0.15, respectively). In contrast, patients who died between days 30–180 after discharge were more likely to have had an emergency readmission (13.3% vs. 7.2%, *p* = 0.024, respectively). The mortality at 180 days according to emergency readmission status is presented in Fig. 2.

## Discussion

In this study, examining the risk of readmission in patients undergoing major high-risk emergency abdominal surgery, we found that 31.9% and 47.8% of patients were readmitted within 30 and 180 days, respectively. Stomal placement was an independent risk factor for 30-day readmission, and an ASA score of 3 was an independent risk factor for both 30- and 180-day readmission.

Previous studies reporting readmission among patients undergoing emergency abdominal surgery found a 30-day readmission rate of approximately 15%. This rate is much lower than the one reported in this study; however, the majority of existing literature examined readmission after emergency general surgery (EGS), predominantly low-risk procedures such as herniotomies, laparoscopic appendectomies, and cholecystectomies. In contrast, this study only included patients who underwent *high-risk* abdominal surgery such as emergency laparotomies and high-risk laparoscopy (i.e., repairment of perforated ulcers or adhesiolysis due to bowel obstruction). Patients undergoing high-risk emergency abdominal surgery are often old, are suffering from comorbidities, and a degree of frailty, which, combined with a potent immunological and inflammatory surgical stress response, causes organ dysfunction and compromised recovery, leaving patients susceptible to postoperative complications and loss of physical function [16–18]. In current literature, procedure invasiveness is correlated to readmission rate [1, 3, 7, 13, 19], supporting our results. In addition, other studies also found an accumulative risk of readmission beyond the first 30 days after discharge [3, 4, 7].

We found that comorbidities (high ASA score and pulmonary comorbidity) were significantly associated with emergency readmission. Furthermore, an ASA score of 3 was an independent risk factor for both short- and long-term readmission. These findings agree with previous research, indicating that comorbidities are risk factors for readmission [4]. In contrary to our expectation, we did not find a significantly increased risk of readmission in patients with ASA scores 4 or 5. Nevertheless, our cohort included very few patients with ASA scores above 3, and we assume that a small sample size prevents us to find a true association.

The main reasons for short-term readmissions were wound complications and complications related to the gastrointestinal tract, such as prolonged paralysis, high-output stoma, and abdominal pain directly related to the surgery. This is in agreement with previous research, which reported the main reasons for 30-day readmission were surgical complications such as gastrointestinal or wound complications [1, 2]. Furthermore, we found that stomal placement during the index procedure is an independent risk factor for short-term emergency readmission. Stoma placement is often due to more severe abdominal emergencies. Most likely, they vastly contribute to the number of readmissions due to prolonged paralysis and volume depletion due to high-output stoma. This is supported by previous research, which showed that the creation of an ileostomy is a pronounced surgical risk factor for readmission, with studies reporting a 30 percent readmission rate within 30 days, most often due to dehydration, surgical site infection, pain, or small bowel obstruction [20].

The reasons for long-term readmissions were a broad range of medical conditions, particularly infections, cardiovascular and thromboembolic causes, constipation, and unspecified abdominal pain. In line with this, Havens et al. reported that older patients (> 65 years of age) undergoing major abdominal surgery were more often readmitted due to cardiovascular complications, volume depletion, infections, or malnutrition than younger patients (< 65 years of age) [1]. This study only examined the first 30 days after discharge; to our knowledge, no other studies have examined the reasons for readmission after major emergency abdominal surgery beyond the first 30 days. Even so, studies on long-term consequences after abdominal surgery have reported a 20 percent risk of recurrent small bowel obstruction [21], and a 20–40% risk of chronic abdominal pain after open abdominal surgery [22].

We found that the 30-day mortality was lower among patients with readmissions compared to patients without readmissions, and the opposite was observed long-term. This was surprising; however, the sample size was small, and might be an incidental finding. Nevertheless, we speculate that immediate emergency readmissions are often due to easily treatable conditions (wound infections, constipation, insufficient pain relief). In contrast, later readmissions are due to more severe conditions (infections, cardiovascular disease, thromboembolic events), which is supported by our findings.

This study has some limitations. First, due to our sample size, some patient characteristics subgroups were small, which may prevent us from finding associations. Second, the patient population is a heterogeneous group with severe comorbidities and many emergency readmissions, probably not all related to the index procedure/illness. This may have masked a true association. Nonetheless, readmissions unrelated to the index procedure/illness were excluded from this study. Third, we had no information on emergency contacts made in the primary sector (general practitioner), which might have underestimated our results. However, contacts handled in the primary sector are most likely minor, such as insufficient pain relief and minor wound complications, as all severe complications are referred to the hospital. Fourth, information on socioeconomic status was not available in our cohort, which previously has been found to contribute to readmission.

Nevertheless, this study is strengthened by its prospective design and detailed information on patient characteristics, pre-, peri-and postoperative factors, and date of death. Furthermore, we had the fortuity to include all emergency hospital readmissions, regardless of LOS and contact reason, eliminating the risk of missing less severe postoperative complications, and are thereby able to uncover the extent of unplanned readmission after surgery. In this study, there was no loss to follow-up during the six-month follow-up from discharge. Because of the detailed information from the patient records, we had the opportunity to exclude irrelevant contacts, which otherwise could have distorted the results.

This prospective study is the first to examine short- and long-term readmission rates and causes of emergency readmission after major emergency abdominal surgery. The results of this study suggest that the risk of out-of-hospital complications and readmission is increased beyond the immediate postoperative period and that the reasons vary with time, meaning that an individualized post-discharge follow-up for risk patients could increase days at home for some patients. In agreement with recent literature, most factors related to readmission in this study are non-modifiable (ASA score, stomal placement etc.). Still, when zooming in on the specific mechanism behind each readmission, we find that they often are multifactorial and in several instances, preventable [8, 24].

Furthermore, this study adds an European angle on risk factors for readmission. Most current studies are retrospective registered based and examine short-term readmission in a US setting, where care fragmentation, insurance coverage, and access to care are proven factors affecting post-discharge outcomes after emergency abdominal surgery [23]. These conditions are not translatable to most European countries with public health care and easy hospital access.

In conclusion, we found that the risk of emergency readmission within 180 days of discharge following major emergency surgery was almost 50%. The significant risk factors for readmission were stomal placement and ASA score, and the main reasons for short-term emergency readmission were directly related to the surgery. In contrast, long-term emergency readmission was due to various reasons, such as infections, cardiovascular and thromboembolic reasons. This study supports that patients undergoing major emergency abdominal surgery suffer from ill health long-term, and for some, even permanently. More research is needed to develop tools to optimize the patient in the postoperative period and after discharge from the hospital.

### Supplementary Information

Below is the link to the electronic supplementary material.

Supplementary file1 (DOCX 21 KB)
