# Supplementary material for: Short and long-term readmission after major emergency abdominal surgery: a prospective Danish study
Source: Eur J Trauma Emerg Surg. 2023 Aug 30;50(1):295–304. doi: 10.1007/s00068-023-02352-3 (PMC10923996; doi:10.1007/s00068-023-02352-3)
Supplement: Supplementary file 1 — Supplementary file1 (DOCX 21 KB) [file 68_2023_2352_MOESM1_ESM.docx]

| **Table S1. Independent risk factors for any emergency readmission** | | | | | | |
| --- | --- | --- | --- | --- | --- | --- |
|  | 30-day follow-up | | |  | 180-day follow-up | |
|  | HR^a^ (95% CI ^b^) | | p-value |  | HR^a^ (95% CI ^b^) | p-value |
| Male *(ref = female)* | 1.07 (0.79-1.46) | | 0.66 |  | 0.94 (0.72-1.21) | 0.64 |
| Age *(ref = <60 years of age)* |  | |  |  |  |  |
| 60-69 | 0.79 (0.49-1.28) | | 0.34 |  | 0.73 (0.49-1.08) | 0.12 |
| 70-79 | 0.93 (0.60-1.44) | | 0.75 |  | 0.86 (0.60-1.24) | 0.42 |
| ≥ 80 | 0.92 (0.54-1.59) | | 0.77 |  | 0.97 (0.62-1.51) | 0.88 |
| WHO Performance Status *(ref = PS 0)* |  | |  |  |  |  |
| 1 | 1.05 (0.72-1.54) | | 0.78 |  | 1.18 (0.86-1.61) | 0.33 |
| ≥2 | 1.01 (0.62-1.64) | | 0.97 |  | 1.26 (0.85-1.88) | 0.30 |
| ASA score *(ref = ASA 1-2)* | | |  |  |  |  |
| 3 | 1.61 (1.11-2.33) | | 0.013 |  | 1.44 (1.06-1.95) | 0.002 |
| 4–5 | 1.86 (0.83-4.18) | | 0.14 |  | 1.61 (0.83-3.11) | 0.07 |
| Current smoker *(ref = no)* | | 0.79 (0.54-1.17) | 0.24 |  | 0.96 (0.71-1.31) | 0.82 |
| Body Mass Index *(ref = BMI 18,5-25)* | | |  |  |  |  |
| <18,5 | 1.13 (0.58-2.18) | | 0.72 |  | 1.14 (0.67-1.95) | 0.63 |
| 25-30 | 0.78 (0.53-1.15) | | 0.21 |  | 0.88 (0.64-1.20) | 0.42 |
| >30 | 1.01 (0.69 -1.49) | | 0.95 |  | 0.96 (0.69-1.33) | 0.79 |
| Active cancer *(ref = no)* | 1.22 (0.82-1.82) | | 0.32 |  | 1.31 (0.94-1.83) | 0.12 |
| Procedure *(ref = surgery without bowel resection)* | | |  |  |  |  |
| Upper gastrointestinal surgery | 1.32 (0.76-2.30) | | 0.33 |  | 1.21 (0.77-1.90) | 0.41 |
| Bowel resection with anastomosis | 1.27 (0.74-2.18) | | 0.39 |  | 1.05 (0.65-1.69) | 0.85 |
| Bowel resection with stoma | 1.51 (1.02-2.23) | | 0.04 |  | 1.30 (0.95-1.79) | 0.10 |
| Other ^c^ | 0.86 (0.34-2.18) | | 0.75 |  | 0.56 (0.24-1.29) | 0.17 |
| Complication *(ref = no)* | 0.93 (0.62-1.41) | | 0.71 |  | 1.09 (0.79-1.51) | 0.59 |
| Reoperation *(ref = no)* | 0.01 (0.70-1.47) | | 0.50 |  | 1.13 (0.82-1.58) | 0.46 |
| Destination at discharge *(ref = home without rehabilitation)* | | |  |  |  |  |
| Home with rehabilitation | 0.99 (0.66-1.52) | | 0.99 |  | 1.07 (0.77-1.49) | 0.69 |
| Rehabilitation facility | 1.31 (0.75-2.28) | | 0.34 |  | 1.02 (0.64-1.63) | 0.94 |
| Other ^d^ | 1.64 (0.83-3.23) | | 0.15 |  | 1.34 (0.73-2.46) | 0.35 |
| Transferred other department *(ref = no)* | 0.97 (0.57-1.64) | | 0.91 |  | 1.05 (0.69-1.61) | 0.82 |
| Length of stay *(continuous)* | 0.99 (0.98-1.01) | | 0.57 |  | 1.00 (0.99-1.02) | 0.56 |
| ^a^Hazard ratio. ^b^95% Confidence Interval.  ^c^Splenectomies, appendectomies, cholecystectomies and patient who were transferred to another hospital for final surgery.  ^d^Nursing home or hospice. | | | | | | |
